# Nitrogen enrichment leads to changing fatty acid composition of phytoplankton and negatively affects zooplankton in a natural lake community

**DOI:** 10.1038/s41598-019-53250-x

**Published:** 2019-11-14

**Authors:** Gabriele Trommer, Patrick Lorenz, Ameli Lentz, Patrick Fink, Herwig Stibor

**Affiliations:** 1 Ludwig-Maximilians-University Munich, Department II Biology, Aquatic Ecology, Großhaderner Str. 2, 82152 Planegg-Martinsried, Germany; 2 Present Address: Water management office Ansbach, Dürrnerstr. 2, 91522 Ansbach, Germany; 3 University of Cologne, Institute for Zoology, Zülpicher Street 47b, 50674 Cologne, Germany; 4 Helmholtz Centre for Environmental Research, Department River Ecology, Brückstraße 3a, 39114 Magdeburg, Germany; 5 Helmholtz Centre for Environmental Research, Department Aquatic Ecosystem Analysis, Brückstraße 3a, 39114 Magdeburg, Germany

**Keywords:** Limnology, Freshwater ecology

## Abstract

Secondary production in freshwater zooplankton is frequently limited by the food quality of phytoplankton. One important parameter of phytoplankton food quality are essential polyunsaturated fatty acids (PUFAs). Since the fatty acid composition of phytoplankton is variable and depends on the algae’s nutrient supply status, inorganic nutrient supply may affect the algal PUFA composition. Therefore, an indirect transfer of the effects of nutrient availability on zooplankton by changes in algal PUFA composition is conceivable. While the phosphorus (P) supply in lakes is largely decreasing, nitrogen (N) inputs continue to increase. This paper presents data from a mesocosm field experiment in which we exposed phytoplankton communities to increasing N enrichment. As a consequence, the PUFA composition of the phytoplankton community changed. With increasing nitrogen fertilisation, we observed lower quantities of essential PUFAs, together with a decrease in the abundances of the dominant herbivorous zooplankton *Daphnia sp*. Their biomass was significantly correlated with phytoplankton PUFA content (C18:3 ω3, C20:5 ω3, C18:2 ω6). Our data therefore indicate that changes in nitrogen supply, together with the resultant changes in phytoplankton food quality, can negatively affect the secondary production of herbivorous zooplankton by reducing the availability of essential polyunsaturated fatty acids.

## Introduction

Global anthropogenic activities drastically alter nutrient cycles by increasing energy consumption and biomass production^1^ thereby strongly affecting global ecosystem services^2,3^. Since the key nutrients nitrogen (N) and phosphorus (P) are essential components of the biomass of organisms’ and often limit primary production, changes in their biogeochemical flows can have drastic consequences on ecosystem dynamics. The biogeochemical pathways of both elements are to a large degree influenced by anthropogenic activities resulting in increasing amounts of N and P entering the ecosystems by means of waste water, excessive fertiliser application and soil erosion soils^1,4,5^. There has been an increasing effort for several decades to reduce nutrient loads, especially in freshwater systems where P often limits the primary production^6^. Replacing the P compounds in detergents and/or providing purification plants with highly efficient P elimination techniques has resulted in successful P reduction and the reoligotrophication of water bodies^7,8^. However, N loads have continued to increase, since the diffuse N inputs are difficult to control by means of targeted measures. N is much more mobile than P, and its high dispersal potential means that it can easily enter groundwater and atmospheric pools^9^.

In lakes, P enrichment is often clearly visible by an apparent increase in primary production^6^. Furthermore, P enrichment can potentially result in eutrophication characterised by blooms of toxic or undesired algal species, the oxygen reduction of deep waters, and other undesirable consequences^10^. By contrast, N enrichment of fresh waters is often much more inconspicuous. Only in lakes where N availability limits primary production, eutrophication signatures are visible and observed^11–13^. However, in addition to the quantitative effects of increasing primary production, more subtle qualitative effects can occur in terms of community and biochemical composition. For example, N enrichment can favor certain algal groups, such as mixotrophic algae^14^ via an alteration of the bacterioplankton composition^15^. Such changes in algal community composition can result in changes in food quality for herbivorous zooplankton, as not all algal groups are equally well-suited as zooplankton food. Consequently, recent mesocosm experiments with natural plankton communities suggest that N enrichment is accompanied by a lower trophic transfer efficiency^16^. While zooplankton growth decreases with an increasing N supply, the decline is most pronounced in the case of cladoceran zooplankton^16^, which are a particularly important food source for fish. Cladoceran zooplankton, especially *Daphnia* sp., have been thoroughly investigated and several food quality related factors that influence their fitness have been identified^17,18^. These factors are algal size, gelatinous sheaths, and toxicity, but also nutrient stoichiometry and biochemical composition^17–19^. The fatty acid composition of phytoplankton, especially the contribution of polyunsaturated fatty acids (PUFAs), is a biochemical factor that is already well known from laboratory studies for its consequences for *Daphnia* growth^20–22^. There is evidence that the PUFA composition of phytoplankton appears to exert a higher influence on the somatic growth rates and reproduction of *Daphnia sp*. than do stoichiometric effects such as the C:P or N:P ratio of the phytoplankton biomass^23,24^.

There is a general mismatch in nutrient management strategies that recent reoligotrophication continues to reduce the amount of P in a large number of water bodies, whereas the N supply continues to increase. This unbalanced reoligotrophication can have various undesirable effects. Although large quantitative effects from N supply to P limited systems are not expected, qualitative changes on the biomass stoichiometry^14^ or biochemical composition cannot be excluded. We therefore investigated the qualitative effects of increasing N enrichment on biochemical phytoplankton PUFA composition in an already P deficient system. We exposed a natural spring plankton community to a gradient of N enrichment in an enclosure experiment. The experimental N enrichment was conducted over an ecologically meaningful time scale of ten weeks^25,26^ in order to include not only the short-term direct enrichment effects on phytoplankton, but also the subsequent bottom up effects on higher trophic levels.

## Results

### Water chemistry

**Figure 1:**
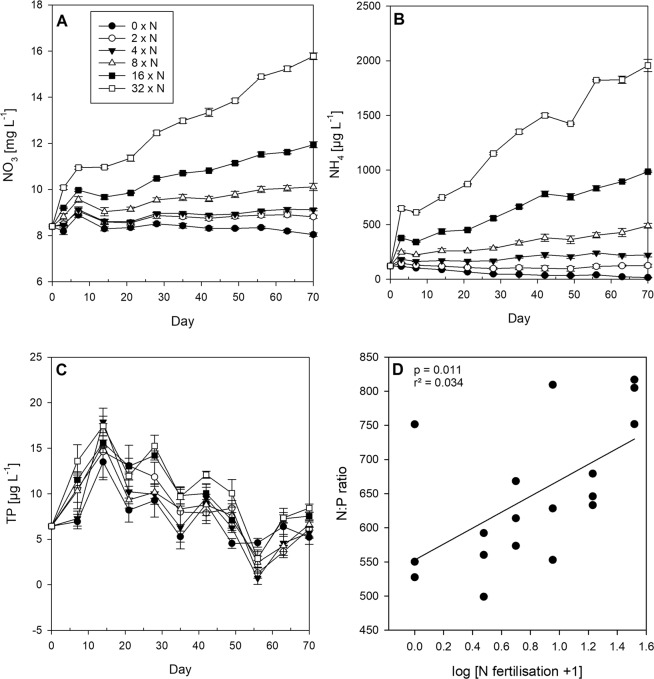
Nutrient development in all six N fertilisation treatments. (**A**) NO_3_ (mg L^−1^), (**B**) NH_4_ (µg L^-1^) and (**C**) TP concentrations (µg L^−1^, mean ± 1 SE of n = 3 replicates) in all six N fertilisation treatments over time. (**D**) Average N:P ratios over time (mean ± 1 SE of n = 3 replicates) against N fertilisation treatment. Significant linear regression line in solid (p < 0.05).

Prior to fertilisation, the nutrient concentrations in Lake Brunnensee were 8.4 mg L^−1^ for NO_3_, 121 µg L^−1^ for NH_4_ and 6.4 µg L^−1^ for TP. Nitrite (NO_2_) was on average 0.09 ± 0.01 (st.dev.) mg L^−1^ over the experimental period. During the experiment, the NO_3_ concentrations ranged from on average 8.4 ± 0.1 mg L^−1^ in the control treatments up to a maximum of 12.5 ± 0.2 mg L^−1^ in the 32 x N treatments. The NH_4_ concentrations ranged from on average 61.9 ± 4.3 µg L^−1^ in the control treatments up to 1169 ± 16.3 µg L^−1^ in the 32 x N treatments. Over the experimental period, the control treatments lost dissolved inorganic N in the form of NH_4_ and NO_3_, whereas in the N supply treatments, NH_4_ and NO_3_ remained at the same level or increased with time in the higher fertilisation levels (Fig. 1A,B). The TP concentration was on average 8.7 ± 4.2 µg L^−1^ over all the treatments and declined on day 56 to 2.7 ± 2.7 µg L^−1^ (Fig. 1C), possibly due to sedimentation loss and microscopic wall growth (periphyton) in the last two weeks. The PO_4_ concentrations were most of the time below detection limit. The silicate concentrations were on average 7.1 ± 0.4 mg L^−1^ in all the treatments and declined at the end of the experiment to ~6.7 ± 0.4 mg L^−1^. According to the experimental design, the average dissolved N:P (based on NH_4_ and NO_3_ to TP) ratio increased significantly with N fertilisation (Fig. 1D).

### Phytoplankton

**Figure 2:**
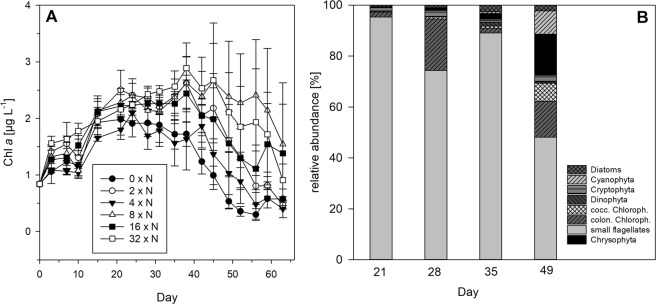
Phytoplankton data in all six N fertilisation treatments. (**A**) Chlorophyll *a* concentrations (µg L^−1^, mean ± 1 SE of n = 3 replicates) in all six N fertilisation treatments over time. (**B**) Relative abundances of the phytoplankton groups in the six treatments as averages per sampling day (differences between the treatments see Table 1).

The total chlorophyll *a* concentration in all the experimental treatments showed a parallel increase from 0.84 µg L^−1^ at the beginning of the experiment to on average 2.2 ± 0.4 µg L^−1^ until day 28 (Fig. 2A). In the mesocosms, maximum chlorophyll *a* concentrations were reached from days 21 to 38. In the control treatment, chlorophyll *a* decreased after day 28, whereas in the higher N treatments chlorophyll *a* started to decrease later after day 40. Higher chlorophyll *a* concentrations with N fertilisation were observed only on day 42 (p = 0.04, r² = 0.24), which is related to the fertilisation design.

Microscopic analyses of the phytoplankton community revealed a flagellate dominated community, comprising chlorophytes and other unclassified small flagellates (which were not further identified). The major taxa from days 21 to 49 were *Chlamydomonas sp*. (on average 10 ± 11%, up to 35%) and unclassified pigmented flagellates of approximately the same size (on average 66 ± 19%, up to 98% on day 21) (Fig. 2B). Colonial chlorophytes (*Botryococcus sp*., *Dictyosphaerium sp*., *Coelastrum sp*., *Gloeocystis sp*., and *Coenochloris sp*.) contributed on average to 10.2 ± 9.3% of the phytoplankton biomass. Cryptophytes were represented by *Cryptomonas sp*. and *Rhodomonas sp*. and comprised on average 1.9 ± 1.8% of the total phytoplankton biomass. Chrysophytes (mainly *Dinobryon sp*.) contributed on average to 4.9 ± 11.9% (up to 16% on day 49) of the phytoplankton biomass. Diatoms contributed on average to only 1.5 ± 1.7% of the phytoplankton community and were represented by the genera *Achnanthes sp*., *Asterionella sp*., *Cyclotella sp*., *Cymbella sp*., *Fragilaria sp*., *Navicula sp*., and *Synedra sp*. Coccoid chlorophytes (2.3 ± 3.3%), dinophytes (0.6 ± 0.7%), and cyanobacteria (2.6 ± 6.0%) represented on average only a minor proportion of the phytoplankton community, with slightly higher abundances on day 49 (Fig. 2B). The statistical analyses revealed a significant relationship between total phytoplankton biovolume and N fertilisation on days 21 and 28, which was due to the large proportion of unclassified small flagellates present (Table 1). On day 49, the biomass of the chlorophyte colonies increased with N fertilisation (Table 1), which was driven by one single mesocosm (No. 18). Relative abundances of chlorophyte colonies did not show any correlation to N fertilisation, only the 2 x N treatment was lower than the control and 32 x N treatment (see Table 1, ANOVA results). No differences between treatments were observed in the other phytoplankton groups on that day (chrysophytes: p = 0.80, r² = 0.00; coccoid chlorophytes: p = 0.16, r² = 0.08; cyanophytes: p = 0.30, r² = 0.07).

**Table 1:** Statistical results of the main phytoplankton groups (>5% abundance of total phytoplankton biomass).

Day, No. observations (n), Degrees of freedom (df)	model		Total biovolume	Chlorophyta colonies	Other flagellates
**21** n = 18 df = 17	Lr ANOVA	p r² p F	**0.02** -0.31	0.39^a^ 0.04 0.35^b^ 5.61	**0.01** -0.38 0.08^b^ 9.73
**28** n = 18 df = 17	Lr ANOVA	p r² p F	**0.01** -0.35	0.07 0.20 0.18 1.84	**0.01** -0.37 0.10 2.44
**35** n = 13 df = 7	Lr ANOVA	p r² p F	0.84 0.00	0.41^a^ 0.06 0.44^b^ 4.81	0.86 0.00 0.47 1.02
**49** n = 18 df = 17	Lr ANOVA	p r² p F	0.32 0.06	**0.02** +0.29 **0.01** 5.31	0.05 0.22 0.80 0.46

Linear regression (lr) of the phytoplankton biovolume and One-Way-ANOVA (ANOVA) of the relative abundances against N fertilisation treatments. Normality and equal variance tests passed if not indicated. Significant results are shown in bold. The leading sign of slope for significant linear regression is indicated (+, -).

^a^Normality test failed.

^b^H-value (not normally distributed: Kruskal-Wallis ANOVA on Ranks).

### Seston stoichiometry

From days 21 to 63, concentrations of particulate organic carbon were on average 0.71 ± 0.28 mg L^−1^, particulate nitrogen 0.17 ± 0.06 mg L^−1^, and particulate P 5.0 ± 2.2 µg L^−1^. The resulting seston C:P ratios fluctuated over the course of the experiment, from a minimum of 173 to a maximum of 710, and were on average 387 ± 97 (see Supplementary Fig. S1). The seston N:P ratios fluctuated from a minimum of 41 to a maximum of 172 and were on average 80 ± 24; the seston C:N ratios ranged from a minimum 2.3 to a maximum of 7.2 (5.0 ± 1 on average, see Supplementary Fig. S1). However, N fertilisation did not significantly affect the biomass stoichiometric ratios, neither as averages over the investigated period (C:P ratio: p = 0.44, r² = 0.04; N:P ratio: p = 0.09, r² = 0.17; C:N ratio: p = 0.24, r² = 0.09) nor on day 49 (C:P ratio: p = 0.39, r² = 0.05; N:P ratio: p = 0.13, r² = 0.13; C:N ratio: p = 0.29, r² = 0.07).

### Phytoplankton fatty acids

**Figure 3:**
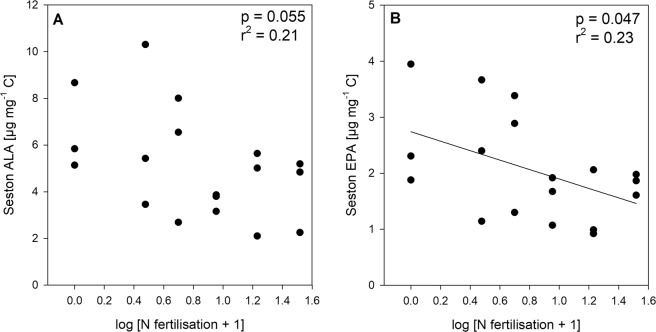
Relationship of fatty acids with N fertilisation treatment. Concentration of (**A**) alpha-linoleic acid (ALA, C18:3 ω3) and (**B**) eicosapentaenoic acid (EPA, 20:5 ω3) against the N fertilisation treatment. Significant linear regression line in solid (p < 0.05).

A total of 32 fatty acids were identified (see Supplementary Table 1). The total fatty acid content indicated no response to increasing N enrichment (p = 0.47, r² = 0.03). However, the fatty acid composition of phytoplankton changed slightly along the N fertilisation gradient (see Supplementary Fig. S2). The total omega-3 PUFAs tended to decrease (p = 0.07, r² = 0.2), while ALA (alpha-linoleic acid, C18:3 ω3) declined substantially with increasing N enrichment (Fig. 3A). Most importantly, the essential PUFA fatty acid EPA (eicosapentaenoic acid, C20:5 ω3) showed a significant negative relationship to increasing N enrichment (Fig. 3B). The total amounts of omega-6 PUFAs and omega-9 monounsaturated fatty acids (MUFAs) did not show a significant relationship to increasing N fertilisation (p = 0.20, r² = 0.10 and p = 0.22, r² = 0.09). Only the relative contribution of omega-9 MUFAs on total fatty acids increased significantly with increasing N fertilisation from 19.1 ± 0.1% in the control treatment to 25.2 ± 0.3% in the 32 x N treatment (p = 0.01, r² = 0.35). On the contrary, the relative contribution the omega-6 PUFAs showed a decreasing trend with increasing N fertilisation (p = 0.06, r² = 0.21).

### Zooplankton

Total zooplankton biomass, including cladocerans, copepods, and rotifers, increased during the experiment from a mean dry mass of 2.9 ± 1.2 µg L^−1^ on day 22 to a mean dry mass of 13.3 ± 8.4 µg L^−1^ on day 63 (see Supplementary Fig. S3). *Daphnia* biomass started to increase on day 35 (see Supplementary Fig. S3). In the control treatment without N fertilisation, *Daphnia* showed the fastest biomass increase and reached the highest biomass (12.9 µg L^−1^, resembling approximately 5 Ind L^−1^) on day 55. In all the other treatments *Daphnia* biomass continued to increase until the end of the experiment on day 63.

**Figure 4:**
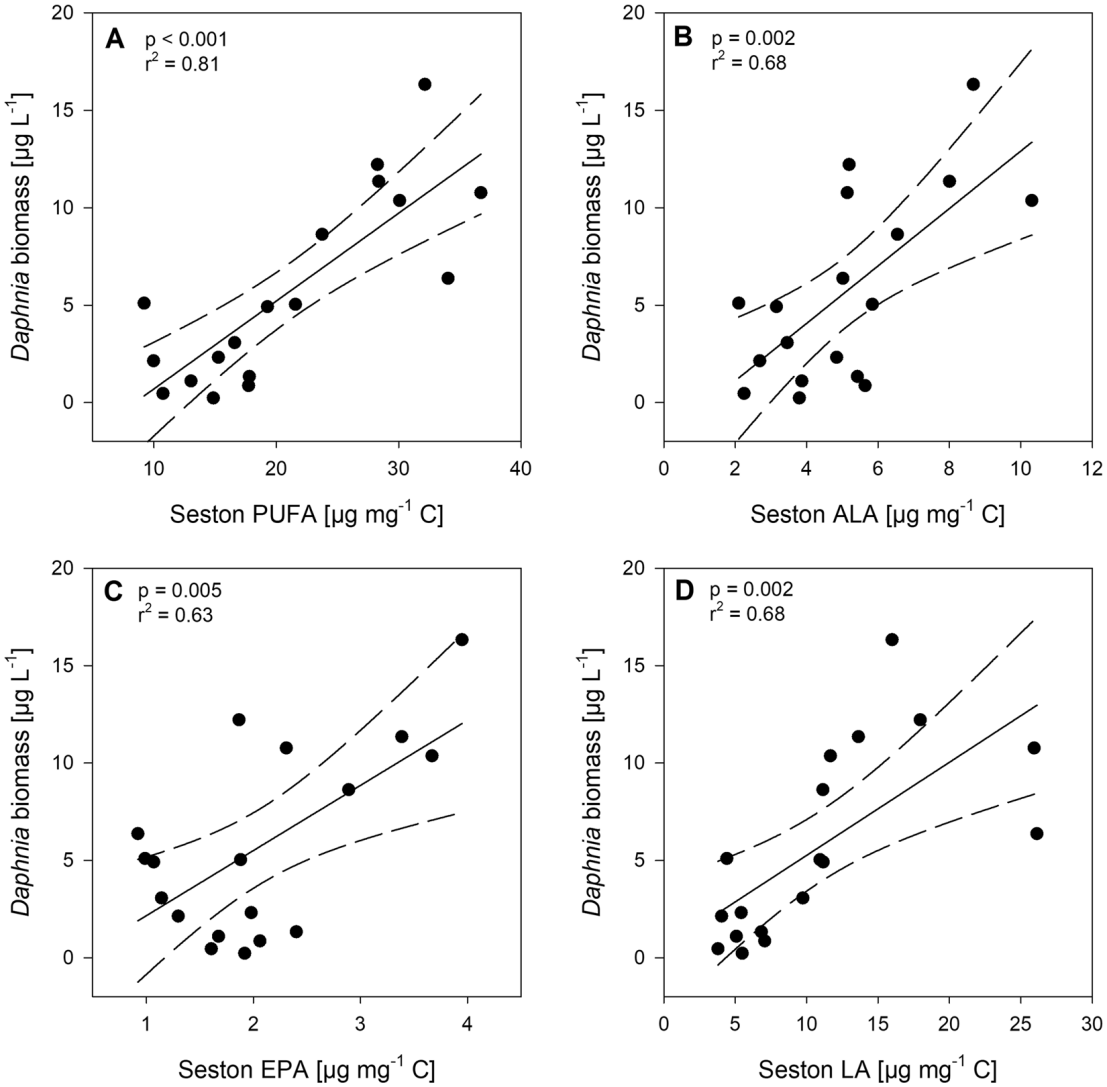
Relationship of *Daphnia* biomass with fatty acid concentrations. *Daphnia* biomass against the concentrations of (**A**) PUFAs, (**B**) alpha-linoleic acid (ALA, C18:3 ω3), (**C**) eicosapentaenoic acid (EPA, 20:5 ω3) and (**D**) linoleic acid (LA, C18:2 ω6). Significant linear regression lines in solid (p < 0.05), 95% confidence interval in dashed lines.

*Daphnia* biomass showed a decreasing trend with increasing N concentrations (day 50: p = 0.10, r² = 0.16), which equals an average decline of 66 ± 38% of *Daphnia* biomass over the experimental N gradient. No relationship between the *Daphnia* biomass and seston stoichiometry could be observed (C:P ratio: p = 0.73, r² = 0.01; N:P ratio: p = 0.23, r² = 0.09; C:N ratio: p = 0.53, r² = 0.03). However, *Daphnia* biomass was strongly positively correlated to the PUFA content in the seston (Fig. 4A). In particular, we found a significant increase of *Daphnia* biomass with ALA, EPA, and LA (linoleic acid, Fig. 4B–D).

## Discussion

Our data indicate that an increase in N supply into the studied lake resulted in a decline of PUFA contents in natural phytoplankton communities. Such a reduction of PUFA contents in natural phytoplankton communities can lead to a lower *Daphnia* biomass. The significant shifts in phytoplankton fatty acid composition caused by increased N enrichment became visible in lower quantities of EPA and ALA, which are known to be key PUFAs in freshwater food webs^20–22^. It had already been demonstrated that the PUFA yield in natural phytoplankton communities is negatively correlated with the P concentration and trophic status across lakes^27^, and is additionally influenced by multiple other environmental stressors such as temperature, light or brownification^28–31^. In this study, we demonstrate that N load can also affect the PUFA composition of natural lake phytoplankton communities. It is well known that algal taxonomic groups differ in their PUFA composition^32^. EPA for example is a major FA of diatoms^33,34^ and ALA for chlorophytes^33^. However, the negative relationship of EPA and ALA with increasing N fertilisation in our study can not be sufficiently explained by changes in the phytoplankton communities. With increasing N fertilisation, we found a weak positive relationship with chlorophyte colony biomass (but not relative abundances) and no relationship with diatoms (both biomass and relative abundances), which would allow this conclusion. Biomass and relative abundances of other unidentified flagellates were also not related to N fertilisation during the period of the *Daphnia* biomass increase, although we cannot fully exclude taxonomic differences within this group. Therefore, we assume that the shifts in PUFA composition in our study were independent of larger taxonomic changes in the phytoplankton community. We find no evidence in our experiment that the reduction in PUFAs with N enrichment was due to a succession of algal groups with different PUFA contents, but was rather dependent on a shift in the PUFA content of the phytoplankton cells *per se*.

Laboratory studies^35–38^ have demonstrated that N deprivation can trigger higher lipid production in green algae, which were also an abundant component of the natural communities in our enclosures. Biochemically, the expression of PUFA biosynthesis genes is upregulated by N depletion^37^. Nitrogen depletion usually increases algal lipid synthesis and the abundance of lipid-related transcripts^37^. In our experiment under conditions of semi-continuous N enrichment, the lipid synthesis appears to be repressed to some extent, leading to lower phytoplankton PUFA contents. In particular, the omega-3 and -6 PUFAs decreased, while the omega-9 MUFAs gained relative importance with N enrichment. Our results provide first evidence that N enrichment not only affects fatty acid dynamics in laboratory strains of cultured microalgae, but also in natural phytoplankton communities. While our study focused on phytoplankton responses, one could potentially also expect such effects on functionally similar microalgal-periphyton. Given the importance of omega-3 and -6 PUFAs in the dietary quality of algae for higher trophic levels, this has potentially far reaching consequences for entire lake food webs.

In our field experiment, the *Daphnia* biomass correlated strongly with the PUFA content of the phytoplankton community and not with seston stoichiometry. This relationship indicates a clear transfer of N enrichment effects through the food web via N-enrichment dependent changes in phytoplankton PUFAs. Despite the natural variation associated with the phytoplankton and zooplankton communities, the relationship between N, *Daphnia* population growth, and the ALA and EPA content of phytoplankton became clearly visible. This is even more remarkable considering the relatively low N enrichment in our study, in comparison to previously described N enrichment field experiments (0.33 mg L^−1^ N per week in the highest 32 x N treatment compared to e.g. 6 mg L^−1^ N per week^39^). The phytoplankton-zooplankton-dynamics indicate that *Daphnia* increased earlier (after day 35) in the control treatment, and that this was followed by an earlier chlorophyll *a* decrease due to grazing, than was the case in the higher N treatments. These dynamics indicate that higher N enrichment and presumably lower PUFA concentrations may lead to worse growth conditions for *Daphnia* in spring, and subsequently could result in a delayed clear-water-phase (after day 50 in treatments 8–32 x N). Poor growth and recruitment of zooplankton under low PUFA conditions was already suggested to have a negative feedback on the control of phytoplankton biomass^40^. To our knowledge our experiments are the first supporting this idea with natural plankton communities. Nitrogen enrichment affected phytoplankton food quality by bottom-up effects but also phytoplankton population dynamics by negative feedback effects of zooplankton grazing on phytoplankton with low food quality. This emphasizes the general importance of PUFAs for pelagic food web dynamics including bottom-up and top-down processes.

Our results suggest that continuous N enrichment and accumulation leads to lower PUFA (especially ALA and EPA) contents, and therefore also to lower food quality of lake phytoplankton. Subsequently, zooplankton and particularly the *Daphnia* biomass will decrease, even if the P concentrations remain unaltered. We can exclude large phytoplankton community composition changes as the reason for the lower *Daphnia* population growth observed, together with the presence of toxic algae or detectable amounts of algae with indigestible gelatinous sheaths. Community shifts towards a lower abundance of EPA (and ALA) rich taxa, such as diatoms and cryptophytes^41^, could in theory cause similar effects to those observed here, which would cause a reduced phytoplankton PUFA content, with subsequent negative consequences for *Daphnia* sp.

Beside an N enrichment, an increase in P limitation may also change the seston stoichiometric composition or the PUFA composition in phytoplankton. Mineral limitation by P in *Daphnia* sp. is predicted at seston C:P ratios >300^42^, as it was the case in our experiment. While previous studies have demonstrated increasing P limitation with N enrichment^14,43^, we were unable to detect signs of increasing P limitation with increasing N enrichment, such as increasing seston N:P ratios. Additionally we did not observe any correlation of seston stoichiometry with *Daphnia* biomass, which does not exclude the possibility that this mechanism could operate supplementary. However, our data suggest an N dependent change in the lipid syntheses of phytoplankton *per se*.

N enrichment has recently been shown to be negatively linked to zooplankton and *Daphnia* biomass across three lakes with different trophic status^16^. However, no mechanism has yet been identified that can explain the relationship observed. The effect of N enrichment on phytoplankton PUFA composition found in this study represents a mechanistic and ecologically important link to how N enrichment can affect higher trophic levels, even in lakes where primary production is P limited and the quantitative food web effects of N enrichment are not typically expected. Based on spring phytoplankton communities that are the nutritional base for zooplankton growth early in the season^25,26^, our experiment demonstrates that “non-limiting” nutrients can also have strong trophic effects on food web dynamics and production. By increasing the non-limiting nutrient, N is able to reduce the secondary production of zooplankton in a similar way as reducing the limiting nutrient P would do.

Our experimental system included a typical plankton community (phyto- and zooplankton) that is characteristic of the majority of P deficient temperate lakes, including the full spring succession dynamics of temperate lakes (PEG model^25,26^). The causal relationship described between N enrichment and reduced biochemical food quality might also be relevant to other lake systems, although the qualitative effects of N enrichment could be masked by the quantitative effects of phytoplankton growth in N limited lake systems^39^ and upscaling from mesocosms to lake systems may have its limitations^44^. However, an N related reduction in secondary production could even affect higher trophic levels, such as fish^43^. Cladoceran zooplankton are known to represent the primary food source for planktivorous fish in temperate lakes^45^, and cladoceran production is positively correlated to fish biomass in lakes e.g.^46,47^. Therefore, the extent to which increasing N enrichment, including organic N forms, contributes to poor food conditions for planktivorous fish has to be further evaluated. Given than non-limiting nutrients can also affect food web dynamics and the production of higher trophic levels, they should be considered in management strategies for freshwater lakes in order to understand the potential ecosystem consequences.

## Material and Methods

### Study site and experimental design

The mesocosm field experiment was performed in Lake Brunnensee in southern Germany during the spring of 2015 (March 17^th^ to May 19^th^), and started directly after the ice melting. Lake Brunnensee is an oligotrophic lake with total phosphorus (TP) concentrations of less than 10 µg L^−1^ and nitrate (NO_3_) concentrations of ~8 mg L^−1^. Accordingly, the N:P ratios were >600:1 at the beginning of the experiment, indicating highly P limiting conditions for the primary producers. The zooplankton community of Lake Brunnensee consists predominantly of calanoid copepods (*Eudiaptomu*s sp.) and cladocera (*Daphnia cf. longispina*).

The mesocosms were made of transparent polyethylene foil (4 m deep, 0.95 m in diameter, ~2.84 m³ in volume). They were closed at the bottom and open at the top, where they were attached to a raft anchored in the centre of the lake. Natural phytoplankton and zooplankton communities were enclosed by lowering the mesocosms into the water column and lifting them back to the surface. A total of 18 mesocosms were filled, and transparent coverings were installed above them in order to minimize the influence of natural precipitation, while ensuring natural light penetration.

The increasing N treatments were based on multiple amounts of natural N fertilisation by nitrate and ammonium (0-, 2-, 4-, 8-, 16- and 32-times the concentration in atmospheric wet deposition). The natural atmospheric wet deposition of the region contains an average supply of 75 mg m^−2^ NO_3_ and 25 mg m^−2^ NH_4_ per week (Bavaria regional state office), with on average 25 L m^−2^ of weekly precipitation (German Meteorological Survey). The control treatment (0) received no N fertilisation, the treatments with 2-times the N concentration in atmospheric wet deposition received an equivalent to 150 mg m^−2^ NO_3_ and 50 mg m^−2^ NH_4_ per week, and so forth for the higher fertilisation treatments.

We fertilised the 18 mesocosms using six N treatments (3 replicates, randomly scattered over the experimental rafts) over a period of 10 weeks, with two fertilisations per week to simulate a semi-continuous N supply. The fertilisation solutions comprised nitrate and ammonium in a 1:1 molar ratio (stock solution: 41.1 mg mL^−1^ NaNO_3_, 29.7 mg mL^−1^ NH_4_Cl). A basic P and Si solution (247.2 mg L^−1^ KH_2_PO_4_, 6164.49 mg L^−1^ Na_2_SiO_3_ × 5 H_2_O) was prepared in order to counteract nutrient loss by sedimentation (based on 0.056 µg L^−1^ day^−1^ total P in previous years). The respective amounts of the fertilisation solutions (0, 2, 4, 8, 16, and 32 mL of stock solution) were transferred into labelled 1 L polyethylene bottles for each treatment in the laboratory (Table 2). 10 mL of basic P and Si solution was added to all the bottles, which were then filled with distilled water. The control treatment (0) received only 10 mL of the basic P and Si solution. The nutrient solution was given to each mesocosm out on the lake, and a Secchi disk was lowered twice to ensure the mixing of the added nutrients. In order to ensure different starting conditions for the individual treatments, the first N fertilisation was given on day 2, with four times the common fertilisation amount.

**Table 2:** Experimental design of the applied N fertilisation in each N treatment with given volumes (mL) and respective amounts (mg) of NO_3_ and NH_4_.

Fertilisation amounts of N treatments	0 x N	2 x N	4 x N	8 x N	16 x N	32 x N
NO_3_: mL mg	0 0	2 60	4 120	8 240	16 480	32 960
NH_4_: mL mg	0 0	2 20	4 40	8 80	16 160	32 320

The amounts were given twice a week and in a 1:1 molar ratio.

### Sampling and laboratory analyses

Sampling for chlorophyll *a* occured twice a week, and sampling for water chemistry, phytoplankton, and zooplankton occurred once a week. All water samples for chlorophyll *a*, water chemistry, and phytoplankton, were taken with an integrated water sampler (KC DenmarkA/S research equipment) of between 1 and 3 metres. The water was filtered through a 250 µm gauze to exclude mesozoplankton.

The water chemistry analyses included NO_3_ and NO_2_ measurements, which were performed by ion chromatography (Dionex ICS-1100, Thermo Scientific, USA) after 0.45 µm filtration of enclosure water (CS 400 cellulose acetate syringe filters; Nalgene, USA). The NH_4_ was measured by fluorometry (Trilogy Laboratory Fluorometre Module CDOM/NH4; Turner Designs, USA) using the orthophthalate method^48^. Prior to the measurement, 2.5 mL of mesocosm water was mixed with 10 mL of a working reagent, which included orthophtalate, sodium sulphite, and borate buffer, and was incubated for two hours in darkness. Dissolved inorganic phosphorus (PO_4_) was measured by ion chromatography (Dionex ICS-1100, Thermo Scientific, USA) after 0.45 µm filtration of enclosure water (CS 400 cellulose acetate syringe filters; Nalgene, USA). The total phosphorus (TP) was measured by means of spectrophotometry (Shimadzu UV-1700, Shimadzu Cooperation, Germany) on 12 mL of mesocosm water, using the molybdenum blue method^49^. The silicate concentrations were analysed on April 7^th^ (day 21), April 21^th^ (day 35), May 5^th^ (day 49) and May 19^th^ (day 63). Silicate was analysed by filtering 100 to 200 mL of enclosure water onto cellulose-acetate filters (0.6 μm pore size, Satorius). The filters were subsequently extracted in a water bath (95 °C, for 4 h, in 0.2 mol NaOH)^50^ and were measured spectrophotometrically using the molybdenum blue method.

For the analyses of particulate organic carbon (POC), particulate nitrogen (PN), and particulate phosphorus (PP), 100 mL to 250 mL of enclosure water was filtered onto pre-combusted (4 h, 450 °C), acid washed (10% HCl) glass fibre filters (GF/F; Whatman, USA), which were subsequently frozen (−20 °C). The samples from the biomass maximum to the end of the experiment (April 7^th^ to May 19^th^ 2015) were analysed as being representative of food quality for zooplankton. The measurements for POC and PN were conducted using an elemental analyser (vario Micro cube, Elementar, Germany), after thawing and drying the filters, and compacting them into small tin caps. The PP was measured after sulfuric acid digestion of the filters with a spectrophotometer (Shimadzu UV-1700, Shimadzu Cooperation, Germany) by applying the molybdenum blue method. Afterwards, the stoichiometric ratios of biomass C:N, N:P and C:P were calculated.

In order to follow the phytoplankton development, chlorophyll *a* measurements were performed twice a week *in vivo* using an Algae lab Analyser (bbe Moldaenke, Germany). This device measures the total chlorophyll *a*, and additionally separates the excitation spectra of four pigment groups into the blue, green, brown, and mixed spectral group^51^.

The microscopic counting of the phytoplankton community was conducted on Lugol fixed samples for the biomass maximum (April 7^th^, 14^th^, and 21^st^ 2015) and the biomass decline (May 5^th^ 2015, day 49) (n = 18, except for April 21^st^ 2015 where only 13 samples could be counted). Applying the Utermöhl method^52^, we placed 25 to 50 mL of each sample into sedimentation chambers. The algae were settling down for at least 24 hours and then they were counted under an inverted microscope (Leica, Germany). In the case of large taxa (e.g. *Ceratium hirudinella*), the whole area of the sedimentation chamber was counted using a magnification of 40. Large diatoms, other ciliates and green algae colonies were counted using a magnification of 200, by counting at least two stripes or 100 individuals of the most abundant taxa. Small green algae, small diatoms, flagellates, and heterotrophic nanoflagellates were counted using a magnification of 400, by counting at least two stripes or 100 individuals of the most abundant taxa. The phytoplankton biomass was estimated after measuring the size of 10 individuals of the most abundant taxa, or by using measurements from earlier studies in this lake^53^.

In order to draw conclusions about the fatty acid quality of the zooplankton’s available food, the phytoplankton fatty acid composition was analysed before the increase in zooplankton densities that occurred during phytoplankton biomass decline (on day 50). To this end, 1 to 1.5 L of 250 µm pre-filtered water was filtered onto glass fibre filters (GF/F; Whatman, USA) and submerged in glass vials, together with 5 mL of a 2:1 (vol:vol) mixture of dichloromethane and methanol (chromatography grade).

The phytoplankton lipids were extracted twice with 5 mL of dichloromethane and methanol (2:1), after the addition of 20 µg methyl heptadecanoate and methyl tricosanoate as internal standards for each sample. The resulting extracts were pooled and evaporated to dryness at 40 °C under a stream of N_2_ gas. The lipid residue was then re-dissolved in 5 mL of 3 N methanolic hydrochloric acid (SUPELCO) and the lipid-bound and free fatty acids were transesterified to fatty acid methyl esters (FAMEs) for 20 min at 70 °C. Subsequently, the FAMEs were extracted from the hydrochloric acid using 3 × 2 mL iso-hexane. The hexane supernatants were pooled and evaporated to dryness under a stream of N_2_ gas at 40 °C. The residue was taken up in 100 µL iso-hexane of which 1 µL was injected (splitless) into the inlet (200 °C, 1.5 mL min^−1^ He as carrier gas) of an Agilent 6890 N GC system equipped with a J&W DB-225 fused silica capillary column (30 m, 0.25 mm, 0.25 µm) at 60 °C. The initial oven temperature of 60 °C was held for one minute, followed by a 20 °C min^−1^ temperature ramp to 150 °C, then 7 °C min^−1^ to 220 °C followed by a final 14 minutes at 220 °C as described elsewhere^54^. The FAMEs were quantified via the internal standards and response factors determined for each FAME relative to the standard from mixtures of known composition for details see^54,55^.

The zooplankton sampling was performed once a week from day 22 (April 8^th^ 2015). The samples were taken by slowly hauling up a 105 µm plankton net through a four metre water column in the centre of each mesocosm. Each sample was immediately poured into a 100 mL polyethylene bottle and immediately fixed to a 70% ethanol end concentration. The samples were stored in the fridge (4 °C) until the zooplankton communities were analysed using a stereo microscope (Wild M3Z; Wild Heerbrugg, Switzerland).The specimens were determined to species level if possible. Length measurements were performed by applying an ocular micrometre with a 0.1 mm division scale. The *Daphnia* biomass (consisting of *D. longispina*) was calculated by using the length-weight relationship of Eq. (1):

1 $$\mathrm{Ln}({\rm{w}})=\,\mathrm{Ln}({\rm{\alpha }})+{\rm{\beta }}\,\mathrm{Ln}({\rm{L}})$$

where w = dry weight in µg, L = length in mm, β = regression slope and Ln(α) = intercept. The coefficients Ln(α) = 1.073 and β = 2.89 were chosen for *Daphnia longispina*^56^.

### Statistical analyses

On the basis of our exponential experimental fertilisation design, linear regression models against logarithmic N fertilisation were applied to the response variables (df = 17 unless indicated otherwise). The applied treatment design was tested using linear regression for the average dissolved N:P ratios against logarithmic N fertilisation. In order to analyse the phytoplankton responses, the weekly data of chlorophyll *a* were tested using linear regression models against logarithmic N fertilisation, while the seston stoichiometric ratios (Seston C:P, seston N:P and seston C:N) were tested as an average over the experimental period and on day 49. The responses of microscopic estimated biovolumes of phytoplankton to N treatments were analysed for the most abundant phytoplankton groups (>5% abundance on total phytoplankton) for each day (21 to 49) against logarithmic N fertilisation. In addition, the relative abundances of the individual algal groups (>5% abundance on total phytoplankton) were tested using One-Way ANOVAs for differences between the treatments (with N treatment as the fixed factor). The microscopically estimated total biovolume of phytoplankton was tested using linear regression against logarithmic N fertilisation. The phytoplankton fatty acid composition was tested during chlorophyll *a* decline (indicating strong grazing through *Daphnia*, day 50) as the absolute amounts (PUFA, omega-3 PUFA, omega-6 PUFA, omega-9 MUFA, EPA, ALA, LA) and the relative contribution with linear regression against the N fertilisation treatments. In order to analyse the effects of N enrichment, seston stoichiometry, and biochemical food composition on *Daphnia*, the *Daphnia* biomass on day 50 was analysed using linear regression against the N fertilisation treatments, seston stoichiometry (day 49) and phytoplankton fatty acid composition. The statistical analyses were performed with Systat Software (Sigma Plot 11.0).

## Supplementary information

Supplementary Information
