# Supplementary material for: Nitrogen enrichment leads to changing fatty acid composition of phytoplankton and negatively affects zooplankton in a natural lake community
Source: Sci Rep. 2019 Nov 14;9:16805. doi: 10.1038/s41598-019-53250-x (PMC6856376; doi:10.1038/s41598-019-53250-x)
Supplement: Supplementary file 1 — Supplementary Information [file 41598_2019_53250_MOESM1_ESM.pdf]

Nitrogen enrichment leads to changing fatty acid composition of phytoplankton and negatively affects zooplankton in a natural lake community

Gabriele Trommer, Patrick Lorenz, Ameli Lentz, Patrick Fink, Herwig Stibor

Supplementary Information

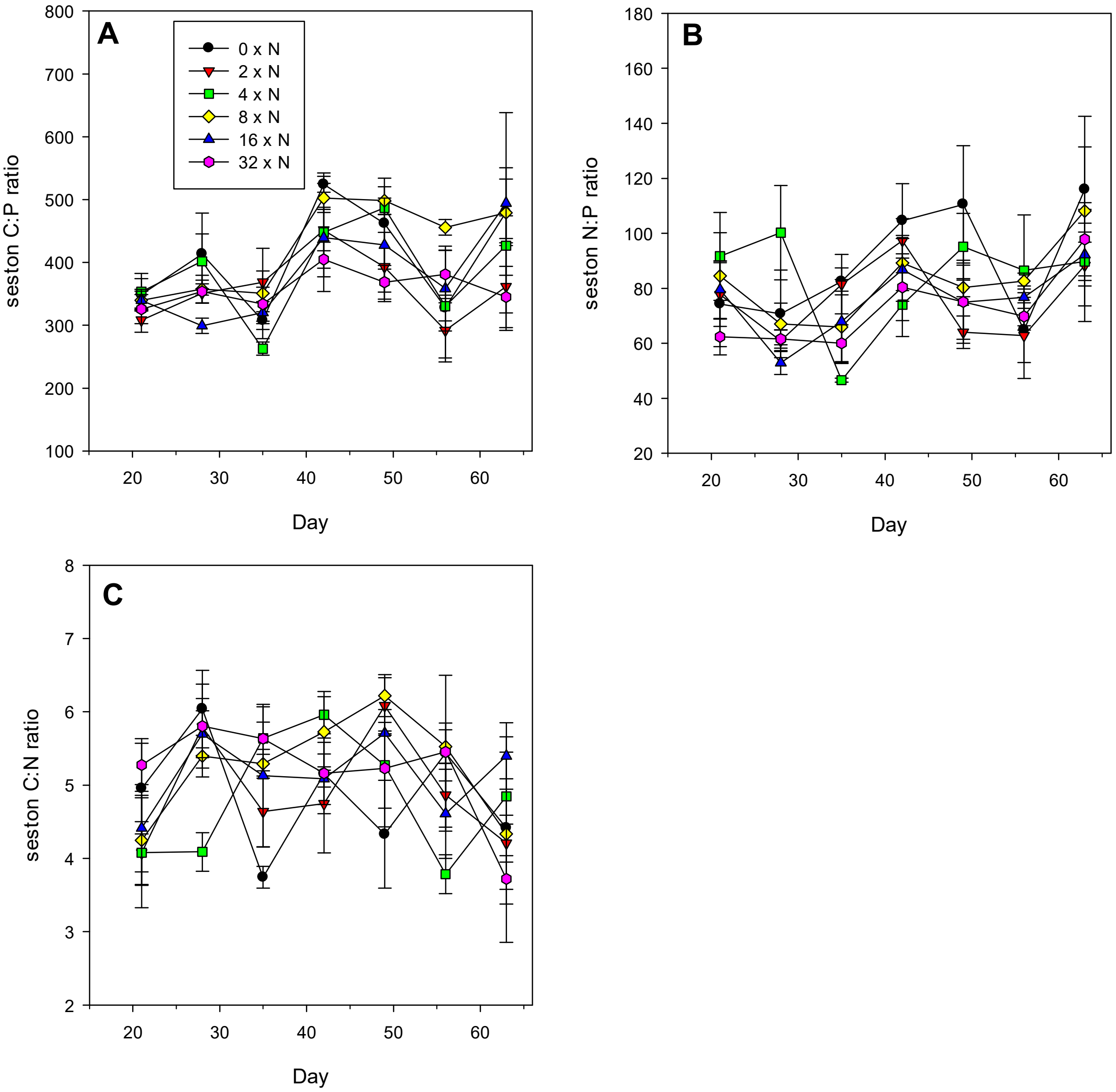

Supplementary Figure S1. Seston stoichiometry in all six N fertilisation treatments. (A) seston C:P ratios, (B) seston N:P ratios and (C) seston C:N ratios (mean ± 1 SE of n = 3 replicates) over time.

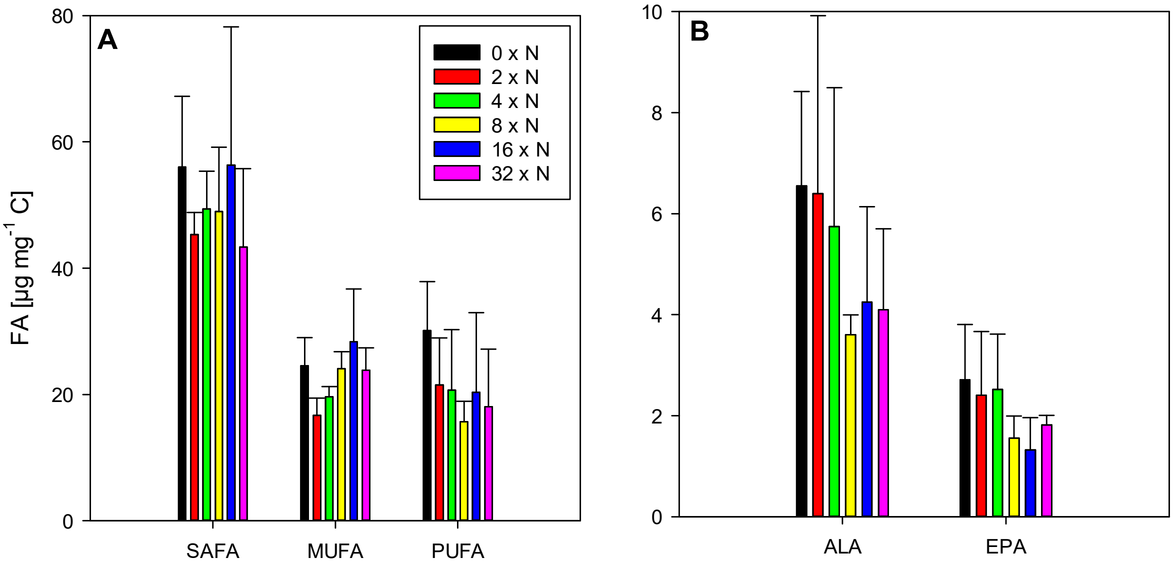

Supplementary Figure S2. Fatty acid composition in all six N fertilisation treatments. (A) Saturated fatty acids (SAFAs), mono unsaturated fatty acids (MUFAs), PUFAs and (B) ALA and EPA (mean ± 1 SE of n = 3 replicates).

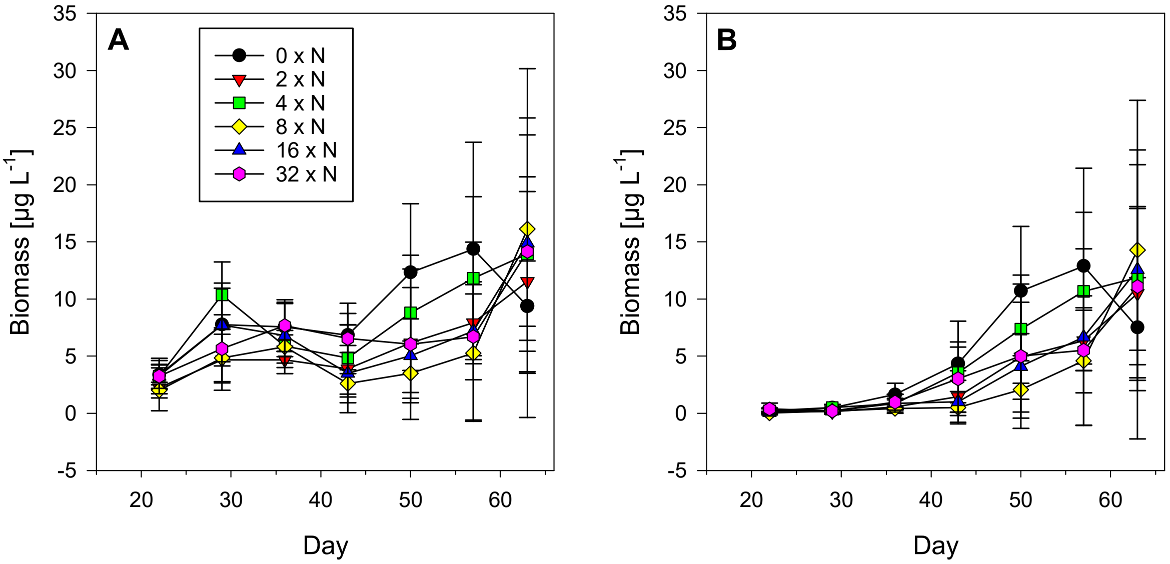

Supplementary Figure S3. Time course of zooplankton biomass in all six N fertilisation treatments. (A) Total zooplankton biomass and (B) *Daphnia sp*. biomass (µg L^{-1}, mean ± 1 SE of n = 3 replicates) in all six N treatments over time.

Supplementary Table 1: Seston fatty acid composition (ng/mg C) of the mesocosms (day 49).

Mesocosm No.	Date	Treatment No.	Treatment N fertilization	Total FA	C10:0	C11:0	C12:0	C13:0	C14:0	C14:1 ω9	C15:0	C15:1 ω9	C16:0	C16:1 ω9	C17:0	C17:1 ω9	C18:0	C18:1 ω9 t	C18:1 ω9 c	C18:1 ω7	C18:2 ω6 t	C18:2 ω6 c	C18:3 ω6	C18:3 ω3	C18:4 ω3
01	06.05.15	1	0	115034,5	0,0	0,0	619,3	292,8	804,6	902,2	823,8	0,0	17424,7	3953,0	903,2	0,0	6180,2	0,0	15474,5	3535,3	0,0	15981,3	0,0	8669,0	691,5
02	06.05.15	1	0	85617,6	0,0	0,0	567,4	270,0	585,3	579,7	696,4	0,0	12767,3	4160,9	592,7	0,0	5526,1	0,0	12129,3	2563,7	0,0	10937,0	317,7	5839,9	463,6
03	06.05.15	1	0	131541,7	0,0	0,0	775,9	0,0	4248,3	707,3	256,1	0,0	23158,6	1111,8	327,6	0,0	7796,3	0,0	23428,6	2832,9	0,0	25933,2	840,8	5139,2	1294,2
04	06.05.15	2	2	76758,8	0,0	0,0	389,6	0,0	422,2	546,8	548,7	0,0	12284,5	1815,5	347,2	0,0	4522,9	0,0	10438,2	1961,3	0,0	9736,8	386,6	3454,6	1042,3
05	06.05.15	2	2	92520,2	0,0	0,0	386,8	0,0	1051,0	940,4	0,0	0,0	14235,7	1392,9	859,6	0,0	5292,1	0,0	12683,2	4093,5	0,0	11670,3	0,0	10305,5	541,2
06	06.05.15	2	2	81308,5	0,0	0,0	0,0	209,4	9087,4	976,6	0,0	514,0	14360,8	1897,1	621,4	0,0	4103,8	0,0	7429,9	2983,8	0,0	6834,6	0,0	5424,8	694,5
07	06.05.15	3	4	92157,1	0,0	0,0	0,0	272,1	704,0	853,3	0,0	513,3	14456,1	1515,5	739,8	0,0	5324,6	0,0	11510,0	3287,8	0,0	11135,7	421,6	6545,6	579,2
08	06.05.15	3	4	102702,8	0,0	0,0	0,0	0,0	731,4	822,5	0,0	487,7	15231,9	1225,3	798,3	0,0	5891,2	0,0	13408,6	2901,0	0,0	13623,5	0,0	8003,1	676,8
09	06.05.15	3	4	74465,4	0,0	0,0	0,0	0,0	7738,3	743,7	206,0	316,8	10169,7	12977,2	359,1	0,0	2421,7	0,0	4833,4	2136,7	0,0	4045,4	275,2	2687,0	668,6
10	06.05.15	4	8	103222,8	0,0	0,0	742,0	424,3	2689,6	491,5	642,6	0,0	11487,6	3248,7	344,9	0,0	5937,5	0,0	13543,8	2080,5	0,0	11159,4	469,2	3155,3	1794,4
11	06.05.15	4	8	88112,9	0,0	0,0	398,2	204,5	8558,2	1310,4	608,5	0,0	12622,4	15462,9	372,5	0,0	3179,6	0,0	6985,4	2752,7	0,0	5487,0	266,5	3797,9	660,0
12	06.05.15	4	8	75109,0	0,0	0,0	356,4	0,0	7676,7	1144,8	615,4	0,0	11224,9	12498,3	336,0	0,0	3055,9	0,0	5843,0	2204,7	0,0	5082,5	247,0	3867,4	588,3
13	06.05.15	5	16	87547,7	0,0	0,0	0,0	0,0	6004,7	1222,2	740,1	0,0	12419,0	10542,7	399,3	0,0	3587,2	0,0	8398,3	2971,2	0,0	7064,2	399,6	5637,2	669,1
14	06.05.15	5	16	74080,8	0,0	0,0	0,0	0,0	8535,7	1334,8	206,2	0,0	11385,1	14219,8	291,5	169,0	3083,9	0,0	5672,9	2190,2	0,0	4415,2	245,2	2100,8	632,5
15	06.05.15	5	16	153407,5	0,0	0,0	726,9	308,7	4579,9	1003,1	1273,3	0,0	24858,1	6699,5	360,9	0,0	10106,2	0,0	25745,7	3452,0	0,0	26124,3	659,8	5014,7	1310,2
16	06.05.15	6	32	113606,1	0,0	0,0	557,8	0,0	550,8	643,5	985,7	0,0	18487,3	5627,7	382,9	0,0	7905,4	0,0	17878,1	3007,0	0,0	17951,8	684,7	5192,5	1136,1
17	06.05.15	6	32	74871,6	0,0	165,2	449,9	194,3	626,9	1152,3	171,8	0,0	11864,8	11984,4	477,1	0,0	3094,0	0,0	7057,8	2840,9	0,0	5419,8	292,5	4840,8	726,9
18	06.05.15	6	32	67359,8	0,0	130,8	0,0	138,1	7852,6	655,1	0,0	256,3	10354,7	12474,0	297,4	0,0	2364,1	0,0	4721,8	2131,3	0,0	3793,4	275,2	2250,4	979,8

Mesocosm No.	Date	Treatment No.	Treatment N fertilization	C19:0	C20:0	C20:1 ω9	C20:1 ω7	C20:2 ω6	C20:3 ω6	C20:4 ω6	C21:0	C20:3 ω3	C20:5 ω3	C22:0	C22:1 ω9	C22:2 ω6	C23:0	C22:6 ω3	C24:0	C24:1 ω9
01	06.05.15	1	0	15533,4	0,0	0,0	0,0	567,1	0,0	1240,1	0,0	1036,3	3947,9	523,6	397,3	0,0	15533,4	0,0	0,0	0,0
02	06.05.15	1	0	10686,4	0,0	0,0	891,2	430,2	0,0	615,7	961,3	321,2	1880,3	393,6	0,0	754,3	10686,4	0,0	0,0	0,0
03	06.05.15	1	0	14076,5	0,0	0,0	498,1	0,0	0,0	880,1	0,0	316,9	2307,4	947,2	588,0	0,0	14076,5	0,0	0,0	0,0
04	06.05.15	2	2	12474,8	0,0	0,0	421,5	0,0	0,0	487,8	0,0	198,9	1142,2	382,3	1129,2	150,3	12474,8	0,0	0,0	0,0
05	06.05.15	2	2	10319,5	0,0	0,0	0,0	0,0	0,0	1462,0	0,0	2423,4	3667,0	341,9	534,8	0,0	10319,5	0,0	0,0	0,0
06	06.05.15	2	2	10343,6	0,0	0,0	0,0	0,0	0,0	1272,3	0,0	1163,1	2401,3	277,2	369,4	0,0	10343,6	0,0	0,0	0,0
07	06.05.15	3	4	14079,4	0,0	0,0	111,9	365,3	0,0	1014,1	0,0	797,0	2889,8	446,5	515,1	0,0	14079,4	0,0	0,0	0,0
08	06.05.15	3	4	15939,6	0,0	0,0	0,0	522,3	0,0	1077,4	0,0	1077,8	3385,7	475,3	483,8	0,0	15939,6	0,0	0,0	0,0
09	06.05.15	3	4	11014,0	0,0	0,0	106,0	0,0	0,0	742,9	0,0	254,7	1298,8	176,5	279,9	0,0	11014,0	0,0	0,0	0,0
10	06.05.15	4	8	18827,8	0,0	0,0	4315,5	0,0	0,0	0,0	0,0	370,4	1070,1	341,8	0,0	1258,3	18827,8	0,0	0,0	0,0
11	06.05.15	4	8	9870,4	379,4	0,0	479,6	0,0	0,0	1457,5	0,0	812,8	1919,0	200,7	0,0	456,4	9870,4	0,0	0,0	0,0
12	06.05.15	4	8	8449,1	0,0	0,0	0,0	0,0	0,0	1010,7	0,0	564,7	1675,3	218,8	0,0	0,0	8449,1	0,0	0,0	0,0
13	06.05.15	5	16	11265,0	565,1	145,8	0,0	290,5	0,0	880,5	0,0	734,2	2061,6	285,2	0,0	0,0	11265,0	0,0	0,0	0,0
14	06.05.15	5	16	8615,4	0,0	0,0	125,0	0,0	0,0	601,3	0,0	220,4	988,8	236,3	195,6	0,0	8615,4	0,0	0,0	0,0
15	06.05.15	5	16	19126,3	0,0	0,0	478,6	0,0	0,0	0,0	0,0	0,0	922,0	934,6	596,5	0,0	19126,3	0,0	0,0	0,0
16	06.05.15	6	32	14044,0	0,0	283,8	230,8	345,3	0,0	714,3	0,0	368,5	1866,7	717,4	0,0	0,0	14044,0	0,0	0,0	0,0
17	06.05.15	6	32	9543,8	0,0	0,0	0,0	311,4	0,0	949,1	0,0	747,3	1979,7	254,5	182,5	0,0	9543,8	0,0	0,0	0,0
18	06.05.15	6	32	7343,4	0,0	0,0	103,5	0,0	0,0	809,1	0,0	224,6	1607,7	218,6	237,1	93,2	7343,4	704,5	0,0	0,0
